# Quantitative Interactor Screening with next-generation Sequencing (QIS-Seq) identifies *Arabidopsis thaliana *MLO2 as a target of the *Pseudomonas syringae *type III effector HopZ2

**DOI:** 10.1186/1471-2164-13-8

**Published:** 2012-01-09

**Authors:** Jennifer D Lewis, Janet Wan, Rachel Ford, Yunchen Gong, Pauline Fung, Hardeep Nahal, Pauline W Wang, Darrell Desveaux, David S Guttman

**Affiliations:** 1 Department of Cell and Systems Biology, University of Toronto, Toronto, ON, M5S 3B2, Canada; 2 Centre for the Analysis of Genome Evolution and Function, University of Toronto, Toronto, ON, M5S 3B2, Canada; 3 Plant Gene Expression Center, USDA, 800 Buchanan St., Albany, CA, 94710, USA

**Keywords:** Next-generation sequencing, yeast two-hybrid, high-throughput screening, Arabidopsis, *Pseudomonas syringae*, type III effector, MLO2, HopZ

## Abstract

**Background:**

Identification of protein-protein interactions is a fundamental aspect of understanding protein function. A commonly used method for identifying protein interactions is the yeast two-hybrid system.

**Results:**

Here we describe the application of next-generation sequencing to yeast two-hybrid interaction screens and develop Quantitative Interactor Screen Sequencing (QIS-Seq). QIS-Seq provides a quantitative measurement of enrichment for each interactor relative to its frequency in the library as well as its general stickiness (non-specific binding). The QIS-Seq approach is scalable and can be used with any yeast two-hybrid screen and with any next-generation sequencing platform. The quantitative nature of QIS-Seq data make it amenable to statistical evaluation, and importantly, facilitates the standardization of experimental design, data collection, and data analysis. We applied QIS-Seq to identify the *Arabidopsis thaliana *MLO2 protein as a target of the *Pseudomonas syringae *type III secreted effector protein HopZ2. We validate the interaction between HopZ2 and MLO2 *in planta *and show that the interaction is required for HopZ2-associated virulence.

**Conclusions:**

We demonstrate that QIS-Seq is a high-throughput quantitative interactor screen and validate MLO2 as an interactor and novel virulence target of the *P. syringae *type III secreted effector HopZ2.

## Background

The Gram-negative bacterial pathogen *Pseudomonas syringae *uses a type III secretion system (T3SS) to translocate type III effector (T3SE) proteins into the cytoplasm of plant cells. The primary function of these T3SEs is believed to be the suppression of plant immunity [1-5]. Some plant hosts are able to respond to this challenge via effector-triggered immunity (ETI), a defense response that is elicited when a plant resistance (R) protein recognizes a specific effector protein either through direct interaction, or indirectly via the action of the T3SE on its host targets [6,7]. The pathogen may respond by acquiring a new effector protein to suppress this recognition or by diversifying away from recognition [7,8]. Thus, the pathogen and host each endeavor to gain the upper hand, resulting in a co-evolutionary arms race.

There are ~60 T3SE families identified in *P. syringae*, yet a majority of these remain functionally uncharacterized. A key to ascribing functions to these virulence proteins will be the identification of their host target proteins. In addition, since many T3SEs have evolved to suppress plant immunity, they can be used as probes to identify important components of resistance signaling pathways.

The HopZ family of T3SE proteins is an evolutionary diverse family that is part of the YopJ T3SE superfamily found in animal and plant pathogens [9,10]. The HopZ family of *P. syringae *is composed of three distinct allele families (HopZ1, HopZ2 and HopZ3), while HopZ1 also has three closely-related allele sub-families (HopZ1a, HopZ1b and HopZ1c). HopZ1a is most similar to the ancestral HopZ allele and is recognized by the ZAR1 resistance protein in *Arabidopsis *[9,11,12]. Although closely related to HopZ1a, HopZ1b is only weakly recognized, and HopZ1c is not recognized in *Arabidopsis *[11]. HopZ2 and HopZ3 are more similar to YopJ superfamily members found in other phytopathogens and were likely acquired by *P. syringae *via horizontal gene transfer [9]. Both HopZ2 and HopZ3 have been demonstrated to enhance *P. syringae *growth on *Arabidopsis *[11,13]. Overall, the HopZ family displays remarkable functional diversification in *Arabidopsis *with members able to enhance bacterial virulence while others trigger ETI. Therefore, the targets of this T3SE family will likely include critical components of host immunity.

The yeast two-hybrid (Y2H) system is a powerful tool to query protein-protein interactions [14,15]. Although several modifications of this method have been developed, they all involve using a bait protein of interest to identify interacting prey proteins. In general, this can be done by using a bait to systematically test specific prey clones, or alternatively using a bait to identify interacting proteins from a pooled library of prey clones. The former method has been applied extensively in high-throughput fashion to generate high quality protein-protein interactome maps [16,17]; nevertheless, the coverage of these interactome maps is relatively low and is typically limited to model organisms, which have high quality libraries of cloned open reading frames (ORFs). If an ORF library is not available, a widely used alternative is to screen a cDNA library; however, this approach carries the ascertainment and representation biases associated with all cDNA library methods. Additionally, screening of these biased libraries is typically limited by the throughput that candidate interactors can be sequenced.

Recently, Vidal and colleagues have established a framework to generate and assess high-throughput Y2H screens and established the first protocol for next-generation sequencing of protein-protein interactomes [18,19]. Their Stitch-seq method employs PCR to concatenate the sequences of putatively interacting bait and prey proteins so that they comprise a single amplicon for downstream next-generation sequencing [19]. PCR stitching is done via common priming sites located downstream of both the bait and prey sequences. Their protocol was specifically designed for Y2H assays using Gateway sequences and clones, but can be generalized to other vectors and a wide variety of interaction assays. While unquestionably promising, there are some potential limitations to this approach. The first relates to the size of the Stitch-seq concatenated amplicon, which is substantially larger than read lengths produced by current next-generation genome sequencers. As the authors note, in principle this obstacle can be overcome as read lengths improve or through paired-end sequencing, but the short reads generated by many next-gen sequencers may prove difficult to associate with a specific gene when working with random cDNA libraries generated from organisms with limited genomic resources. Another potential limitation arises from the long lengths of the stitched PCR products, which encompass the bait ORF, a linker, and the prey ORF, and the need for two rounds of PCR, which may result in PCR biases that influence the recovery of candidate interactors.

Here we describe Quantitative Interaction Screen Sequencing (QIS-Seq), which couples split-ubiquitin yeast two-hybrid screening with Illumina next-generation sequencing to rapidly identify interacting partners of a bait of interest. We employed this high-throughput and quantitative interactor screen to identify host targets of the HopZ family of T3SE proteins, and then demonstrate that these targets include components of plant innate immunity in plants. All members of the HopZ T3SE family (except for HopZ3) are membrane-associated [11,20], and as such, we used the split-ubiquitin yeast two-hybrid screen that utilizes a membrane-associated bait protein [21] in order to enrich for physiologically relevant interactors. We used this approach to identify MLO2 as an interactor of HopZ2, and confirmed this interaction *in vivo *by bimolecular fluorescence microscopy (BiFC). MLO2 has a characterized role in powdery mildew resistance, but had not previously been shown to contribute to *P. syringae *growth. We demonstrate that MLO2 contributes to resistance against *P. syringae *in *Arabidopsis *and is required for HopZ2 virulence function.

## Results

### Evaluation of the cDNA prey library

Our cDNA prey library was commercially made (Norclone Biotech Laboratories, Ontario) from RNA extracted from uninfected 4-5 week old *Arabidopsis *rosettes, as well as plants infiltrated with a virulent pathogen (*P. syringae *pv. tomato DC3000, *Pto*DC3000), a non-virulent strain lacking the T3SS apparatus (*Pto*DC3000 ΔhrcC), and an avirulent strain translocating a T3SE recognized by an *Arabidopsis *R protein (*Pto*DC3000 with AvrRpm1, which is recognized by RPM1). Although it is common to amplify primary cDNA libraries after their initial construction, this step can potentially introduce representation biases that may influence the interactor screen. We amplified the primary library by semi-solid amplification as this method is believed to reduce overall amplification bias [22]. We first sequenced the primary and secondary cDNA prey libraries to assess representation and bias arising from the initial library construction and subsequent amplification. Amplified DNA was sequenced on an Illumina GA-IIx using standard protocols at the University of Toronto Centre for the Analysis of Genome Evolution and Function (CAGEF). Sequencing of the primary- and amplified libraries yielded 59.8 M and 5.8 M reads respectively, which read-mapped to ~11 K *Arabidopsis *genes (4,119 M and 213 M bases of data, Additional file 1). The cDNAs ranged from ~40 nt up to ~2,800 nt, with most not being full length since they were generated by random hexamer priming (Additional file 2A, B). A scatterplot of the hits/locus for the two libraries revealed very high congruence (R^2 ^= 0.96, Additional file 2C), indicating that very little bias was introduced during the amplification process.

**Figure 1:**
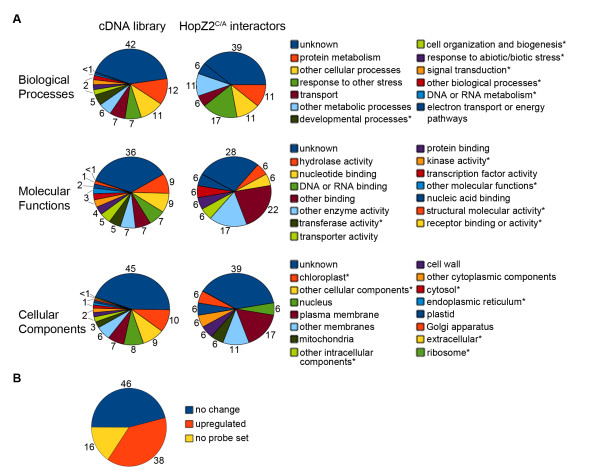
**Characterization of cDNA library and HopZ2^C/A ^putative interactors**. **A**. Percentage of primary cDNA library (left) and HopZ2^C/A ^interactors (right) encoding proteins belonging to gene ontology (GO) terms for biological processes, molecular functions and cellular components. * indicates categories that are missing for HopZ2^C/A ^interactors. **B**. Percentage of genes in library that are upregulated in response to biotic stress (from bacteria, oomycetes or elicitors of innate immunity).

To further evaluate the range of genes represented in the library, we analyzed the gene ontology (GO) annotations of the cDNAs recovered (Figure 1A). Many biological processes were represented including metabolism, response to stress or stimulus, development, transport and signal transduction, although unknown was the most common (42%). The subcellular localization of many of the cDNAs was also unknown; however, loci were identified in virtually every cellular compartment including 13% associated with membranes. Genes with known molecular functions included those involved in protein binding, hydrolase activity, nucleic acid metabolism, transcription factors, transporters, and kinases. We further examined publicly available microarray data available through the CAGEF Bio-Array Resource (BAR, http://bar.utoronto.ca, [23]) to determine whether loci represented in our library were differentially regulated in responses to biotic stress from bacteria (*P. syringae*), oomycetes (*Botrytis cinerea*, *Phytophthora infestans*, *Golonivomyces orontii*), or elicitors of innate immunity (harpins, lipopolysaccharides, and an oomycete elicitor NPP1). 38% of genes were upregulated more than 2-fold in response to biotic stress while 46% did not respond to biotic stress (Figure 1B). A further 16% did not have probe sets to detect transcriptional changes arising from biotic stress. None of our library loci were downregulated in response to biotic stress.

### Quantitative Interaction Screen Sequencing (QIS-Seq)

**Figure 2:**
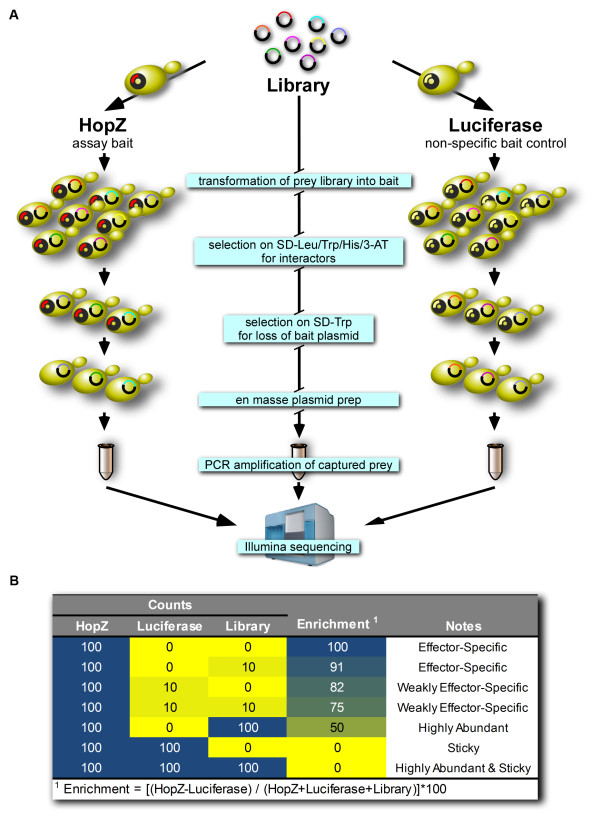
**Schematic of QIS-Seq protocol**. **A**. Outline of QIS-Seq screening protocol described in text. **B**. Example of enrichment calculation showing the effect of varying the recovery of a bait of interest (HopZ), the non-specific control (luciferase), and the frequency in the library. The counts and enrichment score are heat-mapped with high numbers in blue to low numbers in yellow.

Since most members of the HopZ family of T3SE proteins are membrane-associated by myristoylation [11], we adapted the split-ubiquitin yeast two-hybrid system that was developed for transmembrane bait proteins (Figure 2A and Additional file 3) [21]. Based on membrane-association studies of K-Ras, we constructed a bait vector with a C-terminal prenylation signal and polybasic sequence in order to stably associate the protein with membranes (Additional file 4) [24,25].

We screened the *Arabidopsis *prey library by cloning catalytic mutants of all five of the HopZ alleles, as well as the *Arabidopsis *R protein ZAR1 that recognizes HopZ1a into our membrane-tethered bait vector (Figure 2A,Additional files 3, 4). We used the catalytic mutants of the HopZ T3SEs in order to prevent processing of putative substrates and potentially stabilize transient interactions that would occur with the active enzyme. This method has been termed inactive catalytic domain capture (ICDC) and has proven successful for stabilizing enzyme-substrate interactions [26,27]. We also screened the prey library with HopF2*_Pto_*, which is a *P. syringae *T3SE that is unrelated, both with respect to function and sequence, to the HopZ family, as well as luciferase as a non-specific protein control since it should not specifically interact with any *Arabidopsis *proteins. We transformed our baits of interest by lithium acetate/PEG transformation and screened them on plates with dropout media lacking histidine, one of the interaction reporter genes. Approximately 2000 colonies of each bait were collected and then restreaked twice on dropout media lacking tryptophan in order to preferentially retain the prey plasmid and lose the bait plasmid thereby reducing the complexity of the plasmid pool for next-generation sequencing [28]. The colonies were harvested en masse, digested with lyticase, and then plasmids were purified using alkaline lysis. Finally, the prey-plasmid inserts were amplified with vector-specific primers using low-cycle PCR to reduce amplification bias and the PCR product was Illumina sequenced. Each bait provided 4.7 M to 33.6 M quality reads (176 M to 2,544 M bases of data, Additional File 1) which were read-mapped to the *Arabidopsis *reference using Novoalign (http://Novocraft.com), which performs base-quality aware, global alignments with affine gap penalties using full implementation of the Needleman-Wunsch algorithm. The number of reads per *Arabidopsis *coding sequence were converted to reads per million in order to normalize across samples.

We then assessed for overall enrichment of each candidate interactor by scaling the number of hits observed between our bait of interest and each candidate interactor relative to the frequency of those candidates in the primary library. The candidates' general 'stickiness' was also assessed by the number of times it was recovered using luciferase, our non-specific bait protein. Specifically, enrichment was calculated as (T3SE-luciferase)/(T3SE+luciferase+library) *100, where each term is scaled as the number of mapped-reads per million (Figure 2B). This enrichment measure scales from 0 to 100 with higher values corresponding to those candidate interactors that do not bind luciferase (are not sticky) and are rare in the library (Additional file 5).

### Functional analysis of HopZ2-interactors

We elected to focus our initial functional study on HopZ2 because it can promote *P. syringae *growth in *Arabidopsis *and also since a preliminary analysis of the data provided the most interesting candidate interactors. Our enrichment analysis of HopZ2^C229A ^(hereafter HopZ2^C/A^) interactors identified several highly overrepresented and specific candidate interactors (Table 1 and Additional file 5). HopZ2^C/A ^interactors were enriched for membrane-associated proteins (28% HopZ2^C/A ^vs. 13% cDNA library; Figure 1A) as well as proteins associated with responses to stress relative to the prey library (17% HopZ2^C/A ^vs. 7% cDNA library; Figure 1A). Based on our sequencing of the cDNA prey library, we could also assess the percent cDNA coverage of each HopZ2^C/A ^interactor in the prey library. For functional analyses we focused on the HopZ2^C/A ^interactors that had: (1) an enrichment value > 90% (33 loci), (2) were represented by clones > 33 amino acids in the cDNA prey library (ie. cDNA > 100 nucleotides) (18/33 loci) and (3) were specific to HopZ2^C/A ^(i.e. not present with an enrichment score of > 50% in other baits tested) (11/33 loci). We hypothesized that these genes would include HopZ2 virulence targets and that their disruption would alter *P. syringae *growth.

**Table 1:** Top HopZ2^C/A ^Interactors

Locus	HopZ2 rpm^1^	Luciferase rpm	Library rpm	HopZ2 Enrichment^2^	% cDNA Coverage	Additional Interactor^3^	Library^4 ^or T-DNA Line^5 ^Notes
At5g20700*	997.9	0	1.9	99	39		Exon
At4g35450	9,335.5	0	44.7	99	89		5' UTR
At5g13860	2,620.7	0	46.4	98	99		5' UTR
At5g37600	2,531.3	0	51	98	62	HopF2, ZAR1	
At4g35750*	538.7	0	12.2	98	33		Exon
At3g55980	6,583.9	0	189	97	59	Z1b, HopF2, ZAR1	
At3g55410	2,789	0	86	97	32		ND
At1g11310*	1,693.2	0	66.3	96	51		Exon
At3g04790	1,297.5	0	56.4	96	100	HopF2	
At2g34470	3,792.6	1.5	184	95	100	ZAR1	
At4g27520	1,945.6	0	101.2	95	71	Z3, ZAR1	
At3g23890	0.6	0	0.03	95	2	Z3	Short
At3g17820	0.6	0	0.03	95	7		Short
At2g14720	2,792.8	0.5	161	95	37	HopF2	
At2g05160	4,214.3	7.8	265.4	94	65		ND
At1g66200	1.9	0	0.14	93	6	ZAR1	Short
At1g01930	89.7	0.3	7.1	92	60	Z1b	
At1g24706	195.6	0	18.4	91	13	Z1c	
At1g68440*	185.8	0	18.2	91	21		Exon
At1g08980	7,293.2	8	701	91	100	Z1a, HopF2	
At4g00430*	3,886.5	15.3	372.9	91	100		Exon
At2g04140	0.32	0	0.03	90	4	ZAR1	Short
At1g04170	0.32	0	0.03	90	6	Z3	Short
At5g67380	0.32	0	0.03	90	6		Short
At2g26110	0.32	0	0.03	90	4		Short
At5g54890	0.32	0	0.03	90	4	Z3	Short
At5g25110	0.32	0	0.03	90	3	Z1c	Short
At2g22840	0.32	0	0.03	90	5	Z1c	Short
At1g48280	0.32	0	0.03	90	5	Z1c	Short
At3g23390	0.32	0	0.03	90	19	Z1c, HopF2, ZAR1	Short
At5g24010	0.32	0	0.03	90	3	Z1c	Short
At3g55940	0.32	0	0.03	90	2	Z1b, Z1c, Z3, HopF2	Short
At1g66620	0.32	0	0.03	90	5	Z1a, Z1c, Z3	Short

^1 ^rpm = reads per million

^2 ^Enrichment = [(HopZ-Luciferase)/(HopZ+Luciferase+Library)]*100

^3 ^See Additional file 5 for enrichment data with other baits.

^4 ^"Short" indicates that the prey cDNA library clone was less 100 nt in length. ND = not determined.

^5 ^"Exon" & "5' UTR" indicate that the T-DNA insertion is in either the exon or the 5' UTR.

*Tested for increased or decreased bacterial growth

**Figure 3:**
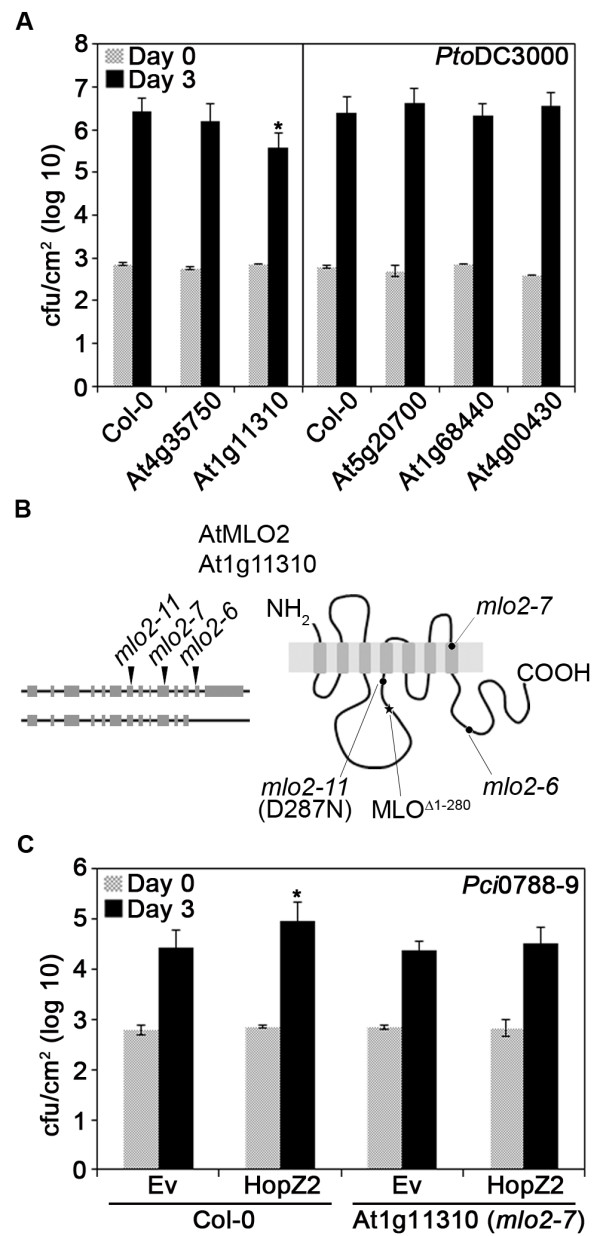
**Disruption of MLO2 compromises *P. syringae *virulence**. **A**. The virulent pathogen *Pto*DC3000 was pressure-infiltrated into the leaves of *Arabidopsis *Col-0 or T-DNA insertion lines in putative interactors of HopZ2^C/A^. An insertion in At1g11310 (*mlo2-7*) results in increased resistance to *Pto*DC3000. * indicates significant difference from Col-0 by Fisher's Protected Least Significant Difference (PLSD) test. Error bars indicate one standard deviation of the mean. **B**. Schematic showing the *MLO2 *gene and protein structure with the point of insertion for the T-DNA insertion lines and the point mutant. The left side shows the gene with introns represented as lines and exons represented as boxes. *MLO2 *has two splice forms which differ at the 3' end of the gene. The right side shows the transmembrane structure of the MLO2 protein. *mlo2-6 *and *mlo2-7 *are T-DNA insertion lines while *mlo2-11 *is a point mutant. MLO^Δ1-280 ^indicates the clone identified in the cDNA library screening. **C**. The weak pathogen *Pci*0788-9 carrying the empty vector (Ev) or HopZ2 was pressure-infiltrated into the leaves of *Arabidopsis *Col-0 or *mlo2-7*. Statistical significance was determined as stated in part A.

We measured *P. syringae *growth in *Arabidopsis *lines carrying T-DNA insertions for each HopZ2^C/A ^specific interactor to determine if the candidate HopZ2 interacting proteins played any role in *P. syringae *disease or resistance. We focused on interactors for which there were confirmed homozygous T-DNA insertion lines available and that were predicted to have the T-DNA insertion in an exon of the gene, and thus be more likely to interrupt the protein (5/33 loci, Table 1). We assayed for changes in immunity by infiltrating the T-DNA insertion lines with the virulent pathogen *Pto*DC3000 and evaluating bacterial growth after three days. Insertions in genes At4g35750, At5g20700, At4g00430 and At1g68440, showed no difference in *Pto*DC3000 growth compared to the wild-type Col-0; however, an interruption in gene At1g11310 (line *mlo2-7*), encoding MLO2 showed a ten-fold decrease in *Pto*DC3000 growth (Figure 3A). To further assess if this locus plays a role in resistance of *Arabidopsis *to *Pto*DC3000 we tested an additional T-DNA insertion line in At1g11310 (*mlo2-6*, Figure 3B). Bacterial growth was reduced by approximately 10-fold in *mlo2-6 *compared to Col-0 wildtype (Additional file 6A) indicating that the *mlo2 *mutation increases resistance to *Pto*DC3000.

The *mlo2-11 *(*pmr2-1*) mutant of *Arabidopsis*, which is a point mutant (D287N) in *MLO2 *Figure 3B), was previously identified in a genetic screen for mutants showing enhanced resistance to powdery mildew [29,30]. However, unlike *mlo2-6 *and *mlo2-7*, but consistent with the previous report [29], *mlo2-11 *(*pmr2-1*) does not exhibit any significant change in *P. syringae *growth compared to Col-0 (Additional file 6B).

### HopZ2 targets *Arabidopsis *MLO2

We further examined whether knocking out MLO2 would affect the virulence advantage normally conferred by HopZ2 when carried by the weak *Arabidopsis *pathogen *P. syringae *pv. cilantro 0788-9 (*Pci*0788-9; [11]). We infiltrated *Arabidopsis *Col-0 and *mlo2-7 *leaves with *Pci*0788-9 carrying either an empty vector (EV) or HopZ2. As previously observed [11], HopZ2 conferred a virulence advantage to *Pci*0788-9 in Col-0, but the HopZ2-associated virulence advantage was compromised in the *mlo2-7 *line (Figure 3C), indicating MLO2 is required for HopZ2-associated virulence.

To determine whether HopZ2 and MLO2 interact *in planta*, we created fusions between both HopZ2^C/A ^and MLO2 to the N- or C-terminus of YFP (nYFP or cYFP) in a glucocorticoid-inducible conditional expression vector. We used a partial clone of MLO2 beginning at amino acid residue 281 of the full-length protein and containing the 4^th^, 5^th^, 6^th ^and 7^th ^transmembrane domains as well as the C-terminal cytosolic tail of the protein (MLO2^Δ1-280^), corresponding to the fragment of the clone in our cDNA prey library (Figure 3B).

**Figure 4:**
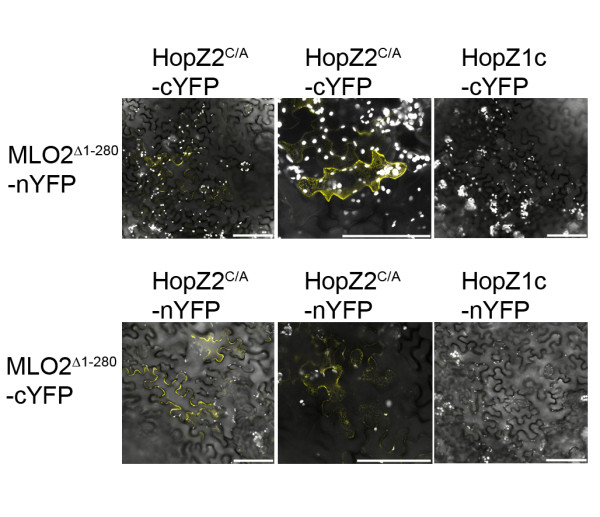
**HopZ2^C/A^, but not HopZ1c, interacts with MLO2^Δ1-280 ^*in planta *by bimolecular fluorescence microscopy**. *Agrobacterium *carrying HopZ2^C/A^::nYFP or MLO2^Δ1-280^::cYFP (or the reciprocal combination) were mixed at equivalent optical densities and pressure-infiltrated into the leaves of *N. benthamiana*. Expression of the proteins was induced by 20 μM dexamethasone. Sections of leaf tissue were imaged with a Leica SP5 confocal scanning microscope 72-96 hours post-induction. The close-up images in the second column show the reticulate pattern reminiscent of the endoplasmic reticulum. The scale bar indicates 100 μm.

We infiltrated equivalent optical densities of *Agrobacterium *carrying HopZ2::nYFP, HopZ2^C/A^::nYFP or HopZ1c::nYFP with MLO2^Δ1-280^::cYFP, as well as the reciprocal combination. We used HopZ1c as a negative control because it did not interact with MLO2 in our yeast two-hybrid screening (Additional file 5, data not shown). Protein expression was induced by spraying the plants with dexamethasone post-infiltration. 72 and 96 hours after dexamethasone application we observed bright fluorescence in leaf sections co-infiltrated with HopZ2::nYFP or HopZ2^C/A^::nYFP and MLO2^Δ1-280^::cYFP, as well as the reciprocal combination (Figure 4; Additional file 7A). No fluorescence was observed with HopZ1c::nYFP and MLO2^Δ1-280^::cYFP (or the reciprocal combination) at these time points. The interaction between MLO2^Δ1-280 ^and HopZ2 localized to the periphery of the cell suggestive of the plasma membrane as well as reticulate network reminiscent of the endoplasmic reticulum (ER; Figure 4). This localization pattern was also observed when an MLO2^Δ1-280^::YFP fusion was transiently expressed in *N. benthamiana *(Additional file 7B).

## Discussion

We developed QIS-Seq, a quantitative, high-throughput yeast two-hybrid screening protocol combined with Illumina next-generation sequencing, to identify putative interacting proteins with the HopZ family of type III effector proteins. QIS-Seq provides many significant advances over traditional interactor screens: (1) it eliminates the need to individually sequence each interacting colony while at the same time vastly increasing the number of candidates interrogated; (2) the results are quantitative and therefore amenable to statistical analysis; (3) the method explicitly evaluates the enrichment of each interactor relative to both its presence in the prey library as well as its general (non-specific) stickiness; (4) sequencing of the prey library provides a hereto unprecedented ability to evaluate the cDNA library for complexity and completeness; (5) it is amenable to any type of yeast two-hybrid screen; (6) it is amenable to any type of next-generation sequencing; (7) it is completely scalable and therefore applicable to experiments run in a very small, multiplex format, to very large automated, high-throughput screens; and (8) the quantitative nature of the data also enhances the method's 'portability' among laboratories.

A number of these points are worth elaborating. The ability to interrogate putative interactors relative to their presences in the prey library (points 3 and 4) is particularly critical when not working with well-established model systems. One of the great benefits of next-generation sequencing is the ability to more easily study non-model systems. By definition, these systems have few established genomic resources, such as normalized cDNA libraries. The *in silico *normalization provided by QIS-Seq facilitates the use of any prey library, regardless of its means of preparation. For example, tissue, cell, age or stage-specific libraries could be rapidly constructed and tested without the need for tedious and sample consuming normalization steps.

Portability in the context mentioned in point 8, means that standards can be established for experimental design, data collection, and data analysis, which will allow experimental results to be comparable among laboratories. Examples of such portability standards included the MIAME (Minimum Information About a Microarray Experiment) [31] and MIGS (Minimum Information about a Genome Sequence) specifications [32]. Another benefit of these standards is that it encourages the development of data repositories and meta-analysis tools such as the Bio-Array Resource [23] for microarray data.

A potential criticism of QIS-Seq is its cost-effectiveness, since the cost of next-generation sequencing is not trivial. Currently, it cost between US$1000-US$4000 for a single channel of Illumina next-generation data (depending on the specific protocol and platform). While we sequenced to quite high coverage in this proof-of-principle study, this depth is not generally required, and we found that 5 million reads were more than adequate. Since the current Illumina HiSeq2000 platform currently produces over 100 million reads per lane, it should be possible to multiplex as many as 20 samples per channel. Importantly, bar-codes can be directly incorporated onto the primers used to amplify the prey-plasmid inserts, thereby permitting the pooling of independent samples prior to Illumina sample prep. Early pooling of bar-coded samples means that only one sample prep is required for all pooled samples, and consequently, while the cost for a single channel of Illumina data may be US$2500, the cost per sample (if multiplexing 20 samples per channel) would only be US$125. This price is substantially less than the cost for Sanger sequencing 100 clones, and the cost will only continue to drop as the next-generation sequencing technology improves.

Since we had previously shown that HopZ2 confers a virulence advantage to *P. syringae *in *Arabidopsis*, we therefore rationalized that we could use HopZ2 as a probe for the identification of innate immunity components. By conducting QIS-seq screens on all members of the HopZ family, we were able to identify proteins that interact specifically with HopZ2. These HopZ2 interactors were enriched for membrane-associated proteins as well as proteins from genes induced during stress responses, including *Arabidopsis *MLO2. The lack of interaction between MLO2 and the other HopZ family members suggests that the HopZ family has diversified to target different host proteins.

MLO2 has seven transmembrane domains with an extracellular N-terminus and an intracellular C-terminus and is localized to the plasma membrane [33]. HopZ2 is normally present at the plasma membrane and would be ideally localized to interact with MLO2 [11]. Our analysis identified a partial clone of MLO2 starting just prior to the fourth transmembrane domain and including the entire intracellular C-terminus (MLO2^Δ1-280^; Figure 3B). Using MLO2^Δ1-280 ^in BiFC analyses, we demonstrated that HopZ2 and MLO2 interact directly *in planta*. However, the observed fluorescence complementation localized to a reticulate structure reminiscent of the ER as well as the plasma membrane. This localization was also observed with MLO2^Δ1-280^::YFP (Additional file 7B), suggesting that MLO2^Δ1-280 ^may be partially mislocalized.

The *MLO *gene was first identified by map-based cloning in barley from mutants that were resistant to the powdery mildew fungal pathogen *Blumeria *(formerly *Erysiphe*) *graminis *f. sp. hordei (*Bgh*) [34]. However, *mlo*-based resistance in crop species has been employed by plant breeders for decades [35]. As in barley, *Arabidopsis mlo2 *confers increased resistance to a powdery mildew fungal pathogen, *Golonivomyces *(formerly *Erysiphe*) *orontii *[30]. However, it has been reported (with data not shown) that *P. syringae *growth did not significantly differ in *mlo2 *compared to Col-0 [29,30]. Vogel and colleagues [29] evaluated symptom production in *mlo2 *(originally called *pmr2*) point mutants when infiltrated or sprayed with *Pto*DC3000, while Consonni and colleagues [30] tested bacterial growth and symptom production from *Pto*DC3000 in a T-DNA insertion line (SAIL_878_H12; *mlo2-5*) that is inserted towards the end of the 6^th ^exon (Figure 3B). Our growth assays with *Pto*DC3000 in the *mlo2-11 *(*pmr2-1*) point mutant confirmed that it did not exhibit increased resistance to *P. syringae *(Additional file 6B). While *mlo2-11 *(*pmr2-1*), which has a D287N point mutation, in the intracellular loop between the third and fourth transmembrane domains [30], confers increased resistance to powdery mildew, it does not appear to be sufficient to confer increased resistance to *P. syringae*, suggesting that MLO2 differentially contributes to immunity against these distinct pathogens. In contrast, unlike Consonni and colleagues we did observe a significant decrease in *Pto*DC3000 bacterial growth in two independent Salk T-DNA lines in the *MLO2 *gene (*mlo2-6 *and *mlo2-7*), although we did not test their T-DNA insertion line (*mlo2-5*). Our results suggest that MLO2 negatively contributes to resistance against *P. syringae *in *Arabidopsis*, and are consistent with the proposed role of MLO2 as a negative regulator of defenses against oomycete pathogens.

Previous work has shown that MLO is relocalized to a lipid raft-like domain in the plasma membrane upon pathogen attack [36]. MLO2 has also been shown to negatively regulate PEN1-dependent vesicular trafficking to regions of the plasma membrane associated with pathogen entry [7,37,38]. PEN1 is a syntaxin that has been associated with aberrant non-host resistance to the fungal barley pathogen *Bgh*, and is likely part of a SNARE complex involved in vesicular trafficking of defense components [37,39]. When PEN1 is recruited to sites of pathogen attack, it contributes to the rapid formation of papillae, an important component of the innate immunity [38,39]. Our data in conjunction with the prior studies suggest that pathogens may stabilize MLO2 or cause its accumulation at the plasma membrane in order to suppress PEN1-dependent secretion of defense components.

There is precedence in the literature for *P. syringae *T3SEs to target negative regulators of plant immunity. The absence of RIN4 in *rin4 rps2 *plants compromises plant immunity whereas RIN4 overexpression enhances immunity [40]. Interestingly, at least four unrelated T3SEs have been demonstrated to target RIN4, potentially to enhance its role as a negative regulator of plant immunity [40-44]. Plant vesicular trafficking pathways are also targeted by *P. syringae *T3SEs. The T3SE HopM1 induces the degradation of AtMIN7, an ARF guanine exchange factor (GEF) that is involved in vesicular trafficking [45]. Similarly, HopZ2 may stabilize MLO2 in order to prevent the secretion of defense components to the regions of pathogen attack.

## Conclusions

Overall we have demonstrated that QIS-Seq provides a powerful new approach to identify protein interactions using next-generation sequencing. We used this approach to identify *Arabidopsis *MLO2 as a virulence target of the *P. syringae *T3SE HopZ2. Since HopZ2 (as well as other *P. syringae *T3SEs) is membrane localized we used the split-ubiquitin yeast two-hybrid system for interaction screening [11,21,46]. However, QIS-Seq is applicable to any sequencing-based yeast two-hybrid screening method. Furthermore, this approach can be applied to both ORF as well as cDNA libraries. Although we sequenced the interactors of individual baits separately, the use of barcodes will allow the sequencing of pooled baits while maintaining the associations between interacting pairs. This approach will increase the number of baits that can be screened per experiment and decrease the cost of screening individual baits. In addition, as the costs of next-generation sequencing experiments continue to drop, QIS-Seq promises to become a cost-effective alternative to traditional yeast two-hybrid screening approaches.

## Methods

### Bacterial strains and routine culture conditions

*Escherichia coli *and *Agrobacterium tumefaciens *were grown in Luria-Bertani broth, and *Pseudomonas syringae *was grown in King's broth (KB). Antibiotics were used at the following concentrations: for *E. coli*, 50 μg/mL kanamycin, 100 μg/mL ampicillin; for *A. tumefaciens*: 100 μg/mL kanamycin, 100 μg/mL rifamcipin; and for *P. syringae*: 50 μg/mL kanamycin, 50 μg/mL rifamcipin and 50 μg/mL cycloheximide.

### Plant growth conditions

*Arabidopsis *plants were grown with 9 h of light (~130 microeinsteins m^-2 ^s^-1^) and 16 h of darkness at 22°C in Sunshine #1 soil supplemented with 20:20:20 fertilizer. *Nicotiana benthamiana *plants were grown with 9 h of light (~130 microeinsteins m^-2 ^s^-1^) and 16 h of darkness at 22°C in Sunshine #1 soil supplemented with 20:20:20 fertilizer and osmocute.

### Cloning

*Pfu *polymerase (Fermentas) was used for all cloning and all constructs were confirmed by sequencing. For the split-ubiquitin constructs, bait genes were amplified by PCR to contain an in-frame HA epitope, a polybasic region (K6 or K8) and a CAAX box, as well as appropriate unique restriction sites. The bait-HA-K6-CAAX genes were cloned into the pBT3-N vector (Dualsystems Biotech) using *Sfi*I. The bait-HA-K8-CAAX genes were cloned into the pTLB-1 vector (gift of Dr. Igor Stagljar, University of Toronto) using *Nco*I. The orientation of each gene in the vector was confirmed.

For the split-YFP constructs, the HopZ genes or the 3' end of MLO2 were amplified by PCR to contain an in-frame HA epitope and appropriate unique restriction sites. All of the genes for the split-YFP system were cloned using *Xho*I and *Stu*I into pBD-nYFP or pBD-cYFP. pBD-nYFP and pBD-cYFP were modified from pTA7002 [47] to add an HA tag and the N- or C-terminus of YFP between the *Stu*I and *Spe*I sites. The N-terminus of YFP encompasses residues 1-155 while the C-terminus of YFP includes residues 156 to the stop codon.

The constructs used for plant infectivity assays were previously described [11]. In brief, the HopZ allele is expressed under its native promoter and contains an in-frame HA tag.

### cDNA library

Five week old *Arabidopsis *rosette leaves were infiltrated by hand with a needleless syringe with *P. syringae *pv. tomato DC3000 (*Pto*DC3000), *Pto*DC3000 carrying AvrRpm1 or the ΔhrcC mutant of *Pto*DC3000 at an optical density of 0.1 (~5 × 10^7 ^CFU/mL) at 600 nm. Infiltrated leaves were harvested at 4 hpi (*Pto*DC3000, *Pto*DC3000 carrying AvrRpm1, or *Pto*DC3000 ΔhrcC) or 12 hpi (*Pto*DC3000, *Pto*DC3000 ΔhrcC). Uninfiltrated leaves were harvested at 4 pi and 12 hpi. RNA was extracted using Trizol (Invitrogen). mRNA was cloned into the pPR3-N vector (Dualsystems Biotech) using the *Sfi*I sites (Norclone Biotech Laboratories, Ontario) with the NubG at the N-terminus of the prey proteins. The library contained ~2.3 × 10^9 ^clones, with an average size of 1.2 kB and was 90% recombinant. Amplification of the library was carried out by the semi-solid method [22]. 0.5 μL of the primary library in *E. coli *strain DH5α was inoculated into 2× LB broth with 0.3% Seaprep agarose (FMC, Rockland) and 100 μg/mL ampicillin. The inoculated cultures were then incubated in a water-ice bath for 1 hour. Subsequently, the inoculated cultures were incubated at 30°C for 44 hours without shaking. To sequence the primary and secondary libraries, low-cycle PCR amplification was carried out with pPR3-N vector-specific primers and a high-fidelity Taq/proofreading polymerase mix (Fermentas, Burlington). This pool of DNA was sheared and prepared for Illumina sequencing by standard methods.

### Yeast two-hybrid screening

HopZ1a^C/A^, HopZ1b^C/A^, HopZ1c^C/A^, HopZ2^C/A^, and ZAR1^CC ^were expressed under the weak CYC1 promoter in the pBT3-N vector, while HopZ3^C/A ^was expressed under the strong TEF1 promoter in the pTLB-1 vector. AP-4 yeast [48] carrying the bait construct were transformed using the PEG/LiAc method. In brief, yeast carrying the bait construct were subcultured in 300 mL SD-Leu overnight to an optical density of 0.6 at 600 nm. Yeast were washed twice in sterile H_2_O and resuspended in 1.5 mL. Transformations were performed with 1 μg of cDNA library, 200 μL of yeast cells and 600 μL of PEG/LiAc (50% PEG, 120 mM LiAc, 10 μL 10 mg/mL boiled salmon sperm DNA) by the heat shock method at 42°C for 45 min. Yeast were washed twice in sterile H_2_O and plated on SD-LeuTrp and SD-LeuTrpHis + 3-amino-1,2,4-triazole (3-AT). Interacting colonies were identified by growth on SD-LeuTrpHis + 3-AT. The appropriate amount of 3-AT was determined for each bait by testing for growth when transformed with the positive control pFur4-NubI and a lack of growth with pFur4-NubG [48]. Screening was performed until ~2000 interacting colonies were identified. Colonies were restreaked twice on SD-Trp to preferentially lose the bait plasmid [10] and grown at 28°C. Prior to plasmid isolation, colonies were restreaked onto SD-Trp and grown at 28°C. Yeast were harvested en masse in SD-Trp and pelleted at 1000 g for 5 min. The pellet (~ 5 g) was washed in 0.1 M NaPO_4 _pH 7.4 and 1.2 M sorbitol and resuspended in 7.1 mL of lyticase buffer [0.1 M NaPO_4 _pH 7.4, 1.2 M sorbitol, 500 μL lyticase (Sigma), 50 μL of 10 mg/mL RNAseA]. 25 KU of lyticase was resuspended in 0.01 M NaPO_4 _pH 7.4 and 50% glycerol and used immediately. Yeast were incubated in the lyticase buffer overnight at 37°C. The Qiagen alkaline lysis spin kit was used to extract the plasmid DNA. The lyticase buffer was used instead of buffer P1. Volumes of buffers P2 and N3 were scaled up to the typical Qiagen proportions based on the volume of the resuspended pellet (1 vol lyticase buffer: 1 vol P2: 1.4 vol N3). Lysis in the P2 buffer was done for 15 min at room temperature and 15 min at 65°C. Buffer N3 was chilled prior to use. After addition of buffer N3, the yeast were incubated on ice for 20 min. Yeast were pelleted at 14000 rpm for 30 min at 4°C. The supernatant was removed and cleared again by centrifugation at 14000 rpm for 15 min at 4°C. The supernatant was loaded onto multiple Qiagen spin columns to purify the plasmid DNA. The columns were washed with buffer PB and buffer PE. Plasmid DNA was eluted with 50 μL of buffer EB after a 1 min incubation. A second elution was performed with 35 μL of buffer EB after a 1 min incubation. To sequence the putative interactors, low-cycle PCR amplification was carried out with pPR3-N vector-specific primers and a high-fidelity Taq/proofreading polymerase mix (Fermentas, Burlington). This pool of DNA was sheared and prepared for Illumina sequencing by standard methods.

### Illumina Sequencing

Illumina sequencing was performed either with 37 cycle single reads or 72 cycle paired-end reads (Additional file 1) following the manufacturer's protocol on an Illumina GAIIx and pipelined using the GA pipeline v1.4.

### Bioinformatics

Illumina reads were mapped to *Arabidopsis *gene models downloaded from NCBI, using a short read mapping tool novoalign (novocraft.com). From the mapping data, the number of mapped reads and the total length of mapped regions for each gene were determined with in house scripts. Read numbers per gene were further normalized as reads per million (rpm) within each sample and compared among the samples. The enrichment of a specific interactor with a bait of interest was determined by considering the number of reads obtained with the bait and luciferase and normalizing against the abundance of reads for luciferase, the bait and the library (Additional file 5). The percentage of the mapped length was calculated using length of mapped regions and the theoretical length of the gene model. Gene Ontology terms for *Arabidopsis *genes were downloaded from the TAIR website, and assigned to the genes in the cDNA library. Up- or down-regulation of each gene in response to biotic stress was determined from microarray data available through the CAGEF Bio-Array Resource (BAR, http://bar.utoronto.ca, [23]. Biotic stress treatments in the BAR included inoculation with virulent, avirulent and non-host *P. syringae*, inoculation with oomycetes (*Botrytis cinerea*, *Phytophthora infestans*, *Golonivomyces orontii*) and inoculation with elicitors of innate immunity (harpins, lipopolysaccharides, and an oomycete elicitor NPP1).

### Genotyping of T-DNA insertion lines

The following T-DNA insertion lines were used: SALK_060284C (At5g20700), SALK_082464C (At4g35750), SALK_079850C (At1g11310, *mlo2-7*), SALK_050191C (At1g11310, *mlo2-6*), SALK_024490C (At1g68440), SAIL_808_A10 (At4g00430). Genomic DNA was extracted from a leaf of 5-6 week old *Arabidopsis *plants. Primers were designed using the iSct feature in the SIGnAL database. Primer sequences are available upon request. PCR-based genotyping was employed to determine the homozygosity or heterozygosity of the individuals. PCR products were sequenced using Big Dye Terminator 3.1 on an ABI 3730 genetic analyzer.

### Infectivity assays

For infiltration, *P. syringae *was resuspended to an optical density of 0.1 (~5 × 10^7 ^CFU/mL) at 600 nm and diluted to a concentration of 1 × 10^5 ^CFU/mL for growth curves. Plants were infiltrated by hand with a needleless syringe, as previously described [49]. Four disks (1 cm^2^) were harvested, ground in 10 mM MgCl_2_, and plated on KB with rifampicin and cycloheximide on days 0 and 3 for colony counting.

### Statistics

For growth assays, 8-10 plants were used for day 3 counts. Significance was determined using Fisher's Protected Least Significant Difference (PLSD) on the day 3 count data. Error bars indicate one standard deviation of the mean.

### *Agrobacterium *transient expression assays and BiFC

Five-milliliter *A. tumefaciens *GV2260 cultures were grown overnight at 28°C in Luria-Bertani broth with kanamycin and rifampicin. The next day, the cultures were washed twice in induction medium (50 mM MES pH 5.6, 0.5% (w/v) glucose, 1.7 mM NaH_2_PO_4_, 20 mM NH_4_Cl, 1.2 mM MgSO_4_, 2 mM KCl, 17 μM FeSO_4_, 70 μM CaCl_2_, 200 μM acetosyringone) [50], and 3.75 mL was inoculated into 35 mL fresh induction medium to grow overnight. The following day, cultures were spun down, washed twice in 10 mM MES pH 5.6 with 200 μM acetosyringone and resuspended to an optical density of 0.4 at 600 nm. The culture containing the MLO2^Δ1-280^-cYFP plasmid was mixed in equal volumes with a culture containing the HopZ1c-nYFP, HopZ2-nYFP or HopZ2^C/A^-nYFP plasmid. The culture containing the MLO2^Δ1-280^-nYFP plasmid was mixed in equal volumes with a culture containing the HopZ1c-cYFP, HopZ2-cYFP or HopZ2^C/A^-cYFP plasmid. The underside of the leaves of 5- to 7-week-old *N. benthamiana *plants were infiltrated by hand with a needleless syringe. Plants were sprayed with 20 μM dexamethasone (Sigma) 1-2 hours after inoculation. Sections of leaves were imaged with a Leica SP5 microscope using Leica software at 24 hours (YFP fluorescence) or 72-96 hours (BiFC) post-dexamethasone induction.

## Abbreviations

ETI: effector-triggered immunity; MLO: powdery mildew resistance gene O; *Pci*: *Pseudomonas syringae *pv. cilantro; *Pto*: *Pseudomonas syringae *pv. tomato; R: resistance; RIN4: RPM1-interacting protein 4; T3SE: type III secreted effector; T3SS: type III secretion system; ZAR: HopZ-activated resistance

## Authors' contributions

JDL, DD and DSG conceived the experiment; JDL performed the experiments; JW and RF assisted with the split-ubiquitin yeast two-hybrid screening; Y.G. performed the bioinformatics analysis; PF ran the Illumina GAII; HN analyzed microarray data from the BAR; PW provided critical input for GAII sequencing; JDL, YG, DD and DSG analyzed the data; JDL, DD and DSG wrote the manuscript. All authors read and approved the final manuscript.

## Supplementary Material

Additional file 1 **Illumina sequencing of cDNA libraries and interactors**. Table showing the prey cDNA library used for the yeast two-hybrid screening, the number of Illumina cycles, the number of quality clusters, and the number of bases for each bait or the cDNA library. Click here for file

Additional file 2 **Characterization of the *Arabidopsis *cDNA library used in this study**. **A**. Histogram shows the number of *Arabidopsis *genes on log scale plotted against the length of the cDNAs present in the cDNA library. There are no genes present in the 2300-2499 or 2600-2699 ranges. There is one gene present in the 2500-2599 and 2700-2799 ranges. **B**. Histogram shows the number of genes on log scale plotted against the percent coverage of the cDNAs in the cDNA library. **C**. Scatter plot of the genes present in the primary and amplified (secondary) libraries. The R^2 ^value of 0.9567 shows a high congruence between the primary unamplified and secondary amplified libraries. Click here for file

Additional file 3 **Schematic of the split-ubiquitin yeast two hybrid approach**. The N-terminus of the bait protein is compromised of the LexA-VP16 transcription factor (VP16), followed by the C-terminus of ubiquitin (Cub), the bait protein of interest, an HA epitope tag, a C-terminal polybasic region (K6 or K8) and CAAX box. We added the K6 (or K8) and CAAX box sequences to non-transmembrane bait proteins in order to anchor them to membranes and minimize autoactivation. At the N-terminus of the prey proteins is the N-terminal half of ubiquitin with an isoleucine to glycine mutation, which reduces non-specific association with Cub (NubG) [21]. The prey proteins may be membrane-associated or cytoplasmic (not shown). In the case of interaction between a bait and prey, the Cub and Nub portions of ubiquitin are brought into close proximity and reconstitute the full ubiquitin protein. A ubiquitin-specific protease (the "blades") cleaves the VP16 transcription factor, allowing it to translocate to the nucleus and activate the HIS reporter gene. Click here for file

Additional file 4 **Bait and prey vectors used in QIS-Seq**. The bait:HA:K6:CAAX was cloned into the *Sfi*I sites of the pBT3-N vector, in frame with an N-terminal LexA-VP16 (VP16) and the C-terminus of ubiquitin (Cub). Bait protein expression is driven by the weak CYC1 promoter. Stronger bait expression was achieved by cloning the bait:HA:K8:CAAX into the *Nco*I site of the pTLB-1 vector, in frame with an N-terminal VP16 and Cub (not shown). The prey cDNAs were cloned into the *Sfi*I sites of the pPR3-N vector, in frame with an N-terminal ubiquitin (the N-terminus of ubiquitin with an isoleucine to glycine mutation, NubG) [21]. Prey protein expression is driven by the weak CYC1 promoter. Click here for file

Additional file 5 **Rank list of top interactors with HopZ2 and their enrichment with other HopZ alleles, an unrelated T3SE HopF2 and ZAR1**. Table indicates the percentage enrichment with each tested bait for the top HopZ2 interactors in *Arabidopsis*. Click here for file

Additional file 6 **Disruption of *MLO2 *compromises *P. syringae *virulence. A**. The virulent pathogen *Pto*DC3000 was pressure-infiltrated into the leaves of *Arabidopsis *Col-0 or the *mlo2-6 *T-DNA insertion line. * indicates significant difference from Col-0 by Fisher's PLSD test. Error bars indicate one standard deviation of the mean. **B**. The virulent pathogen *Pto*DC3000 was pressure-infiltrated into the leaves of *Arabidopsis *Col-0 or the *mlo2-11 *(*pmr2-1*) point mutant. Error bars indicate one standard deviation of the mean. Click here for file

Additional file 7 **HopZ2 interacts with MLO2^Δ1-280 ^in planta by bimolecular fluorescence microscopy and MLO^Δ1-280 ^localizes to a reticulate network. A**. *Agrobacterium *carrying HopZ2::nYFP or MLO2^Δ1-280^::cYFP were mixed at equivalent optical densities and pressure-infiltrated into the leaves of *N. benthamiana*. Expression of the proteins was induced by 20 μM dexamethasone. Sections of leaf tissue were imaged with a Leica SP5 confocal scanning microscope 72-96 hours post-induction. The scale bar indicates 100 μm. **B**. *Agrobacterium *carrying MLO2^Δ1-280^::YFP was pressure-infiltrated into the leaves of *N. benthamiana*. Expression of the proteins was induced by 20 μM dexamethasone. Sections of leaf tissue were imaged with a Leica SP5 confocal scanning microscope 24 hours post-induction. The scale bar indicates 100 μm. Fluorescence is observed in a reticular pattern reminiscent of the endoplasmic reticulum. Click here for file
