# Supplementary material for: Quantitative Interactor Screening with next-generation Sequencing (QIS-Seq) identifies Arabidopsis thaliana MLO2 as a target of the Pseudomonas syringae type III effector HopZ2
Source: BMC Genomics. 2012 Jan 9;13:8. doi: 10.1186/1471-2164-13-8 (PMC3320541; doi:10.1186/1471-2164-13-8)
Supplement: Additional file 1 — Illumina sequencing of cDNA libraries and interactors. Table showing the prey cDNA library used for the yeast two-hybrid screening, the number of Illumina cycles, the number of quality clusters, and the number of bases for each bait or the cDNA library. [file 1471-2164-13-8-S1.DOCX]

| **Additional file 1 Illumina sequencing of cDNA libraries and interactors** | | | | |
| --- | --- | --- | --- | --- |
| **Bait** | **Prey cDNA Library** | **Cycles^1^** | **Number of**  **Quality Clusters** | **Number of Bases** |
| HopZ1a | Primary | 37 | 6,366,624 | 235,565,088 |
| HopZ1b | Secondary | 37 | 6,689,767 | 247,521,379 |
| HopZ1c | Secondary | 72-PE | 14,024,512 | 2,131,725,824 |
| HopZ2 | Secondary | 37 | 6,032,266 | 223,193,842 |
| HopZ3 | Secondary | 72-PE | 16,739,311 | 2,544,375,272 |
| HopF2 | Secondary | 37 | 4,718,958 | 349,202,892 |
| ZAR1 | Primary | 37 | 7,191,607 | 266,089,459 |
| Luciferase | Primary | 37 | 4,744,283 | 175,538,471 |
| 1^o^ Library^2^ | - | 37 & 72-PE | 32,522,067 | 4,119,092,587 |
| 2^o^ Library | - | 37 | 5,753,787 | 212,890,119 |
| ^1^ PE = paired-ended sequencing.  ^2^ Illumina sequencing of primary prey cDNA library. Data were generated using one channel of 37 cycle sequencing and eight channels of 76 cycle paired-end sequencing. | | | | |
