# Supplementary figures and images for: Quantitative Interactor Screening with next-generation Sequencing (QIS-Seq) identifies Arabidopsis thaliana MLO2 as a target of the Pseudomonas syringae type III effector HopZ2
Source: BMC Genomics. 2012 Jan 9;13:8. doi: 10.1186/1471-2164-13-8 (PMC3320541; doi:10.1186/1471-2164-13-8)

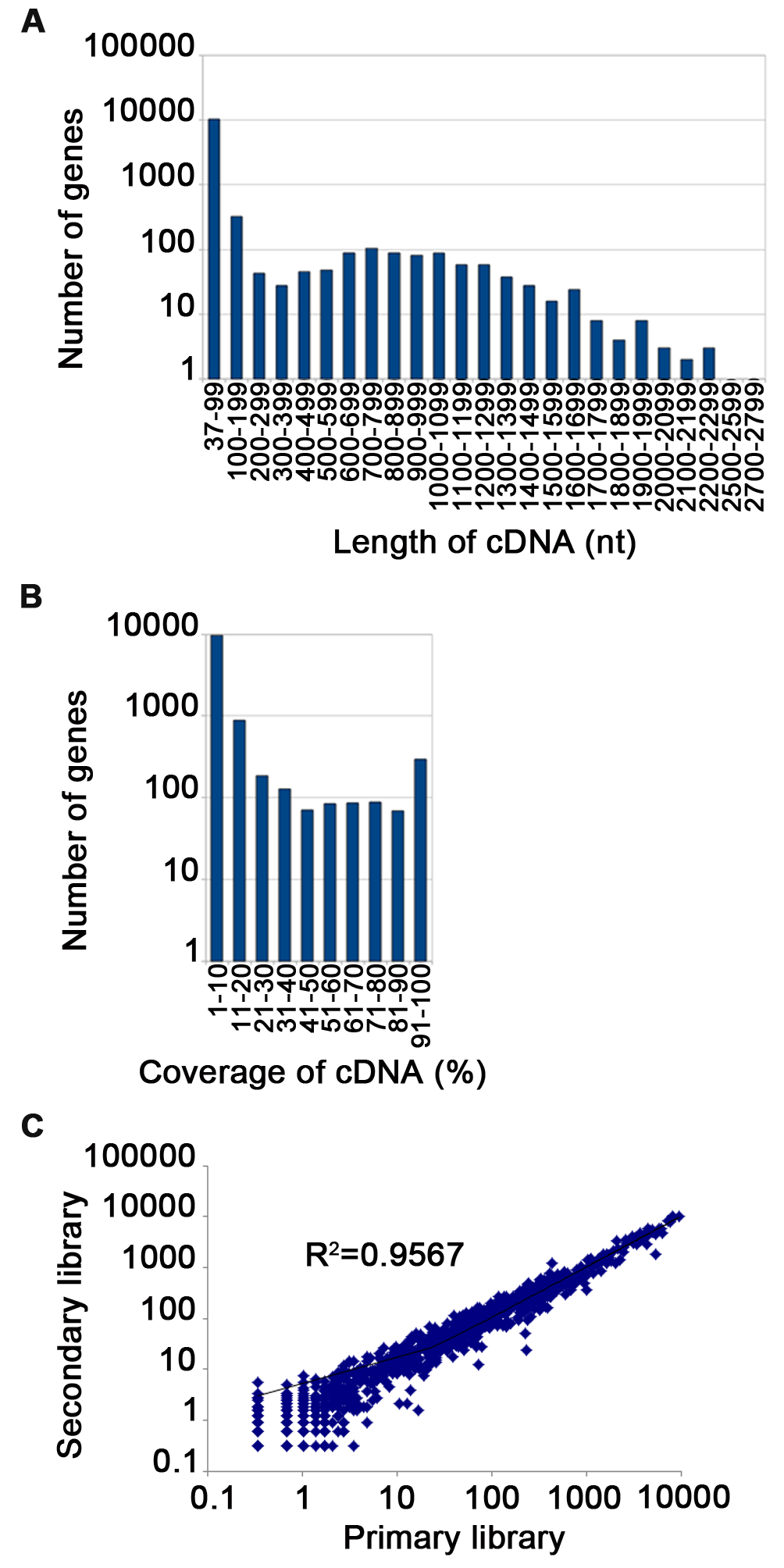

Supplement: Additional file 2 — Characterization of the Arabidopsis cDNA library used in this study. A. Histogram shows the number of Arabidopsis genes on log scale plotted against the length of the cDNAs present in the cDNA library. There are no genes present in the 2300-2499 or 2600-2699 ranges. There is one gene present in the 2500-2599 and 2700-2799 ranges. B. Histogram shows the number of genes on log scale plotted against the percent coverage of the cDNAs in the cDNA library. C. Scatter plot of the genes present in the primary and amplified (secondary) libraries. The R2 value of 0.9567 shows a high congruence between the primary unamplified and secondary amplified libraries. [file 1471-2164-13-8-S2.TIFF]

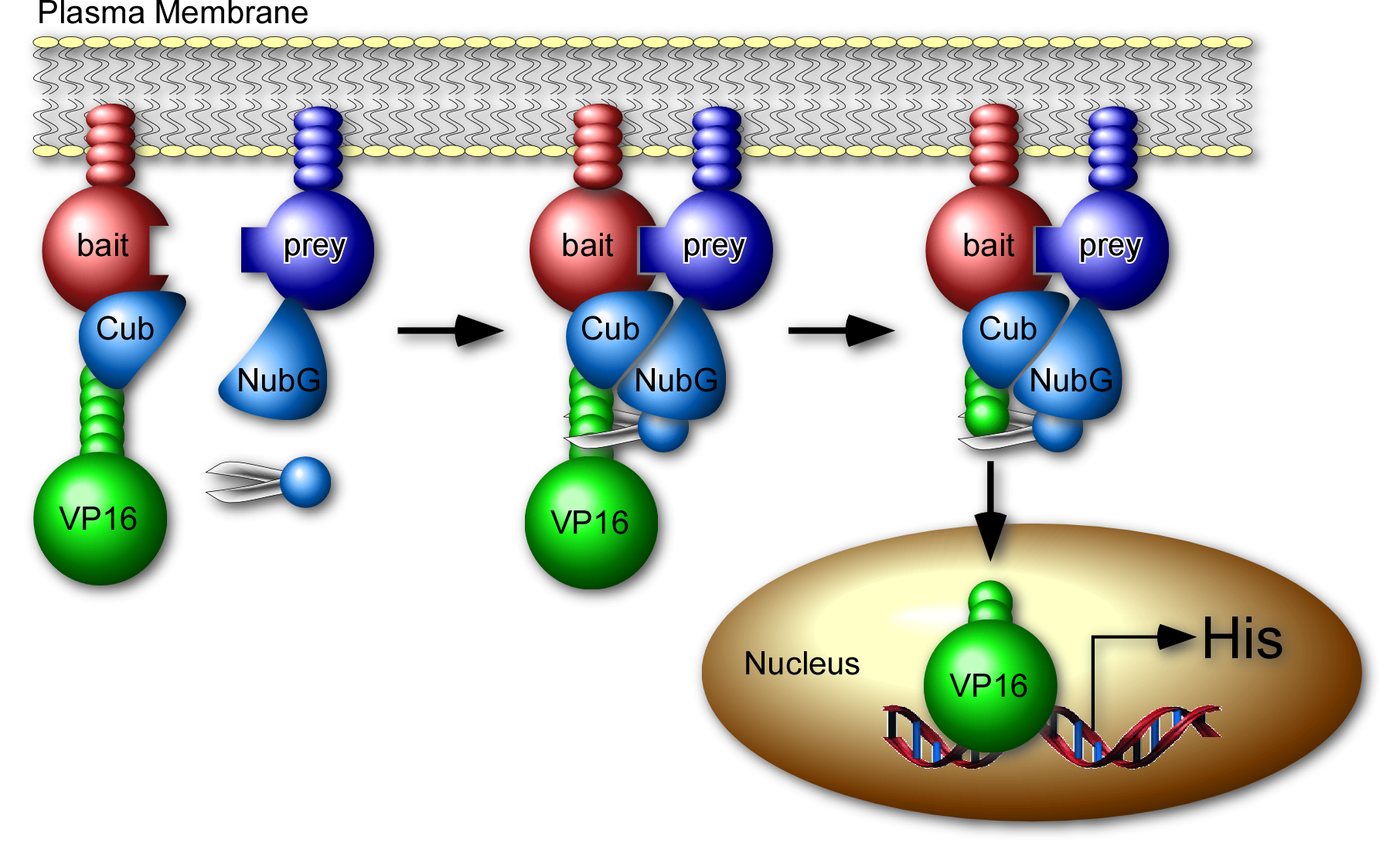

Supplement: Additional file 3 — Schematic of the split-ubiquitin yeast two hybrid approach. The N-terminus of the bait protein is compromised of the LexA-VP16 transcription factor (VP16), followed by the C-terminus of ubiquitin (Cub), the bait protein of interest, an HA epitope tag, a C-terminal polybasic region (K6 or K8) and CAAX box. We added the K6 (or K8) and CAAX box sequences to non-transmembrane bait proteins in order to anchor them to membranes and minimize autoactivation. At the N-terminus of the prey proteins is the N-terminal half of ubiquitin with an isoleucine to glycine mutation, which reduces non-specific association with Cub (NubG) [21]. The prey proteins may be membrane-associated or cytoplasmic (not shown). In the case of interaction between a bait and prey, the Cub and Nub portions of ubiquitin are brought into close proximity and reconstitute the full ubiquitin protein. A ubiquitin-specific protease (the "blades") cleaves the VP16 transcription factor, allowing it to translocate to the nucleus and activate the HIS reporter gene. [file 1471-2164-13-8-S3.TIFF]

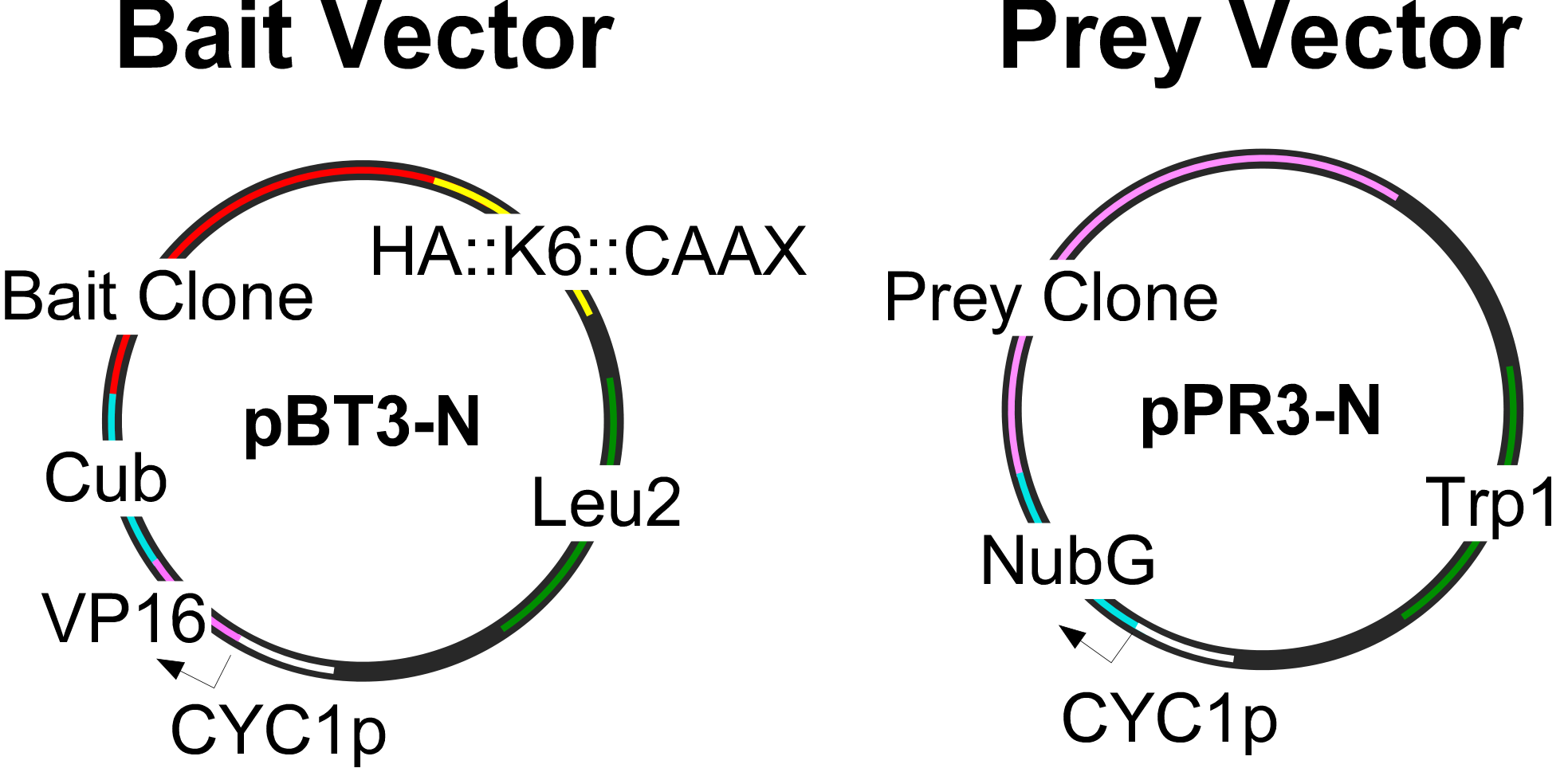

Supplement: Additional file 4 — Bait and prey vectors used in QIS-Seq. The bait:HA:K6:CAAX was cloned into the SfiI sites of the pBT3-N vector, in frame with an N-terminal LexA-VP16 (VP16) and the C-terminus of ubiquitin (Cub). Bait protein expression is driven by the weak CYC1 promoter. Stronger bait expression was achieved by cloning the bait:HA:K8:CAAX into the NcoI site of the pTLB-1 vector, in frame with an N-terminal VP16 and Cub (not shown). The prey cDNAs were cloned into the SfiI sites of the pPR3-N vector, in frame with an N-terminal ubiquitin (the N-terminus of ubiquitin with an isoleucine to glycine mutation, NubG) [21]. Prey protein expression is driven by the weak CYC1 promoter. [file 1471-2164-13-8-S4.TIFF]

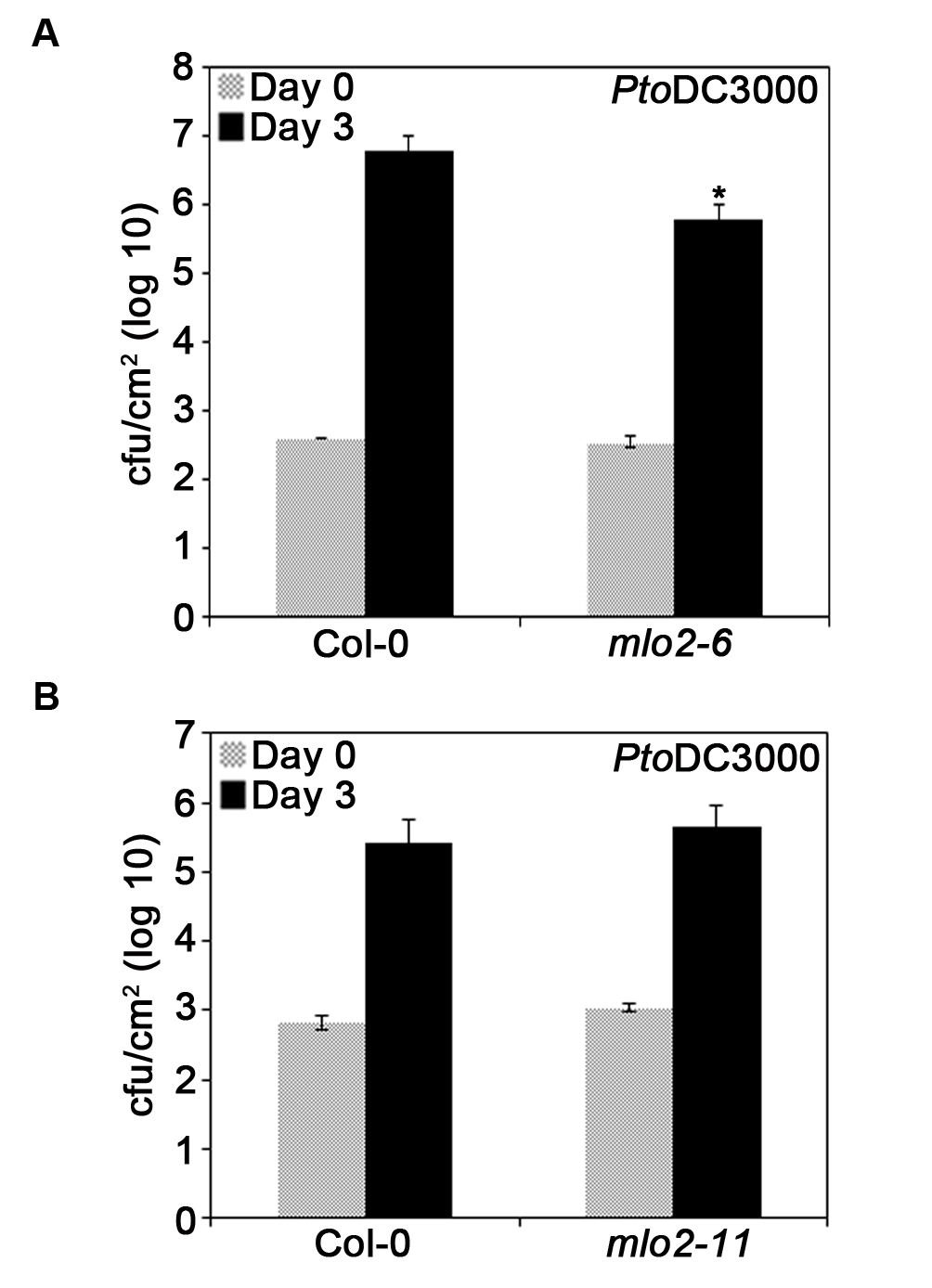

Supplement: Additional file 6 — Disruption of MLO2 compromises P. syringae virulence. A. The virulent pathogen PtoDC3000 was pressure-infiltrated into the leaves of Arabidopsis Col-0 or the mlo2-6 T-DNA insertion line. * indicates significant difference from Col-0 by Fisher's PLSD test. Error bars indicate one standard deviation of the mean. B. The virulent pathogen PtoDC3000 was pressure-infiltrated into the leaves of Arabidopsis Col-0 or the mlo2-11 (pmr2-1) point mutant. Error bars indicate one standard deviation of the mean. [file 1471-2164-13-8-S6.TIFF]

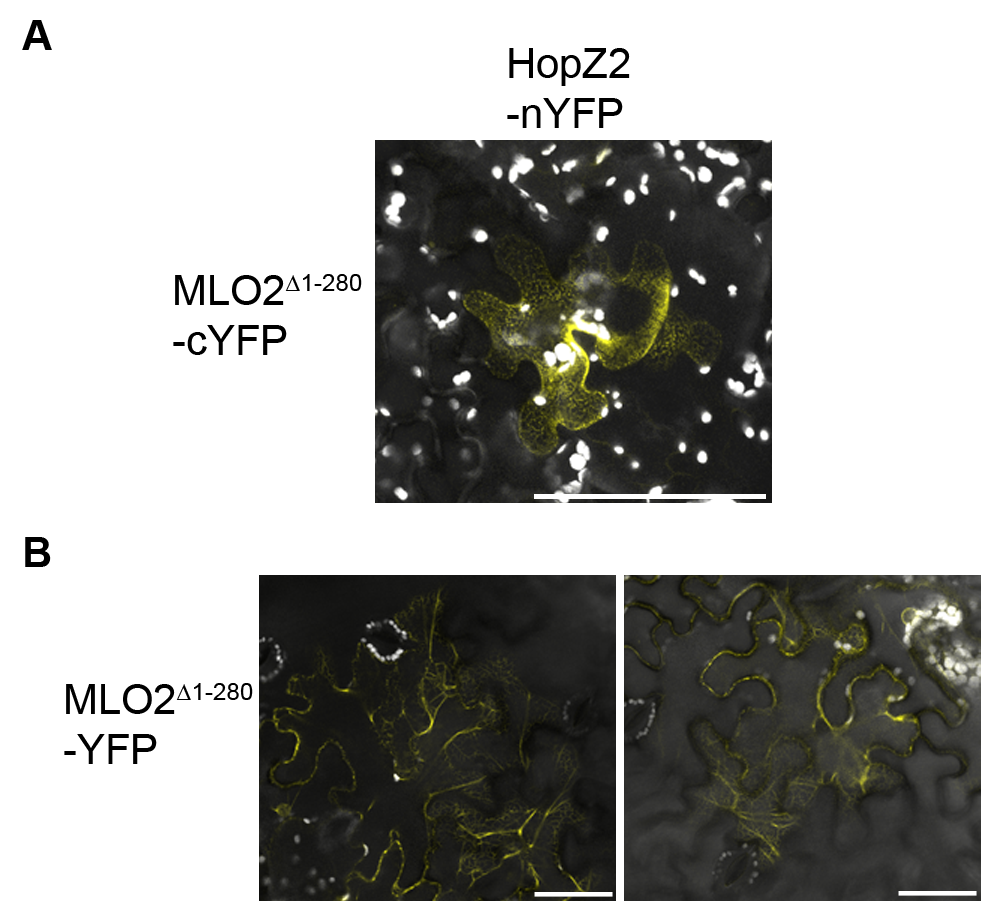

Supplement: Additional file 7 — HopZ2 interacts with MLO2Δ1-280 in planta by bimolecular fluorescence microscopy and MLOΔ1-280 localizes to a reticulate network. A. Agrobacterium carrying HopZ2::nYFP or MLO2Δ1-280::cYFP were mixed at equivalent optical densities and pressure-infiltrated into the leaves of N. benthamiana. Expression of the proteins was induced by 20 μM dexamethasone. Sections of leaf tissue were imaged with a Leica SP5 confocal scanning microscope 72-96 hours post-induction. The scale bar indicates 100 μm. B. Agrobacterium carrying MLO2Δ1-280::YFP was pressure-infiltrated into the leaves of N. benthamiana. Expression of the proteins was induced by 20 μM dexamethasone. Sections of leaf tissue were imaged with a Leica SP5 confocal scanning microscope 24 hours post-induction. The scale bar indicates 100 μm. Fluorescence is observed in a reticular pattern reminiscent of the endoplasmic reticulum. [file 1471-2164-13-8-S7.TIFF]
