# Supplementary material for: Quantitative Interactor Screening with next-generation Sequencing (QIS-Seq) identifies Arabidopsis thaliana MLO2 as a target of the Pseudomonas syringae type III effector HopZ2
Source: BMC Genomics. 2012 Jan 9;13:8. doi: 10.1186/1471-2164-13-8 (PMC3320541; doi:10.1186/1471-2164-13-8)
Supplement: Additional file 5 — Rank list of top interactors with HopZ2 and their enrichment with other HopZ alleles, an unrelated T3SE HopF2 and ZAR1. Table indicates the percentage enrichment with each tested bait for the top HopZ2 interactors in Arabidopsis. [file 1471-2164-13-8-S5.DOCX]

| **Additional file 5** **Rank list of top interactors with HopZ2 and their enrichment with other HopZ alleles, an unrelated T3SE HopF2 and ZAR1** | | | | | | | |
| --- | --- | --- | --- | --- | --- | --- | --- |
|  | **Percentage enrichment with bait of interest^1^** | | | | | | |
| **Gene** | **HopZ2^2^** | **HopZ1a^2^** | **HopZ1b^2^** | **HopZ1c^2^** | **HopZ3^2^** | **HopF2^3^** | **ZAR1^4^** |
| At5g20700 | 99 | - | 13 | - | - | - | - |
| At4g35450 | 99 | - | 2 | - | - | 17 | - |
| At5g13860 | 98 | 1 | 1 | - | - | 16 | - |
| At5g37600 | 98 | 23 | 47 | - | - | 81 | 65 |
| At4g35750 | 98 | - | - | - | - | - | - |
| At3g55980 | 97 | 4 | 76 | - | - | 69 | 90 |
| At3g55410 | 97 | - | - | - | - | 1 | - |
| At1g11310 | 96 | - | - | - | - | 1 | - |
| At3g04790 | 96 | - | 1 | - | - | 78 | - |
| At2g34470 | 95 | - | 1 | - | - | - | 58 |
| At4g27520 | 95 | - | - | - | 60 | - | 60 |
| At3g23890 | 95 | - | - | - | 61 | - | - |
| At3g17820 | 95 | - | - | - | - | - | - |
| At2g14720 | 95 | - | - | - | - | 54 | - |
| At2g05160 | 94 | 2 | - | - | - | - | 43 |
| At1g66200 | 93 | - | - | - | - | - | 62 |
| At1g01930 | 92 | 16 | 98 | - | - | - | - |
| At1g24706 | 91 | - | - | 94 | 3 | - | - |
| At1g68440 | 91 | - | - | - | - | - | - |
| At1g08980 | 91 | 60 | - | - | - | 51 | - |
| At4g00430 | 91 | - | - | - | - | - | - |
| At2g04140 | 90 | - | - | - | - | - | 87 |
| At1g04170 | 90 | - | - | - | 61 | - | - |
| At5g67380 | 90 | - | - | - | - | - | - |
| At2g26110 | 90 | - | - | - | - | - | - |
| At5g54890 | 90 | - | - | - | 61 | - | - |
| At5g25110 | 90 | - | - | 88 | - | - | - |
| At2g22840 | 90 | - | - | 66 | - | - | - |
| At1g48280 | 90 | - | - | 66 | - | - | - |
| At3g23390 | 90 | - | - | 66 | - | 97 | 95 |
| At5g24010 | 90 | - | - | 66 | - | - | - |
| At3g55940 | 90 | - | 89 | 85 | 76 | 92 | - |
| At1g66620 | 90 | 96 | - | 66 | 61 | - | - |
| ^1^ Enrichment = [(Hop-Luciferase) / (Hop+Luciferase+Library)]*100  ^2^ Catalytic cysteine has been mutated to alanine  ^3^ HopF2_Pto_ from *P. syringae* pv. tomato DC3000  ^4^ N-terminal domain of ZAR1 from amino acids 1-144 | | | | | | | |
